# Combined Pimavanserin and Maintenance Electroconvulsive Therapy: A Novel Approach to Parkinson’s Disease Psychosis

**DOI:** 10.7759/cureus.64316

**Published:** 2024-07-11

**Authors:** Riley K Imlay, Majd Alsayed, Madison Starcher, Alfred Tager, James Griffith

**Affiliations:** 1 Psychiatry, West Virginia University School of Medicine, Charleston, USA; 2 Psychiatry, Charleston Area Medical Center, Charleston, USA; 3 Internal Medicine and Psychiatry, Charleston Area Medical Center, Charleston, USA

**Keywords:** atypical psychosis, deep brain stimulator, parkinson' s disease, treatment resistant psychosis, geriatric psychosis, deep brain stimulation. parkinson’s disease, atypical parkinson’s disease, parkinson's disease psychosis, pimavanserin, electroconvulsive therapy (ect)

## Abstract

Parkinson's disease (PD) is among the most common neurodegenerative diseases. Parkinson’s disease psychosis (PDP) is a potential psychiatric manifestation of PD that is associated with increased morbidity and mortality. The treatment of PD with concomitant PDP is challenging as standard-of-care medication to improve motor symptoms can cause or exacerbate PDP. In this case report, we present an atypical presentation of a 70-year-old female who developed PDP only four years after her initial PD diagnosis, much earlier than the established average. Treatment was particularly complex as her PDP symptoms were refractory to PD medication reduction and oral antipsychotics, yet her PD motor symptoms were well controlled with a deep brain stimulator (DBS). We discuss a combination of pimavanserin and maintenance electroconvulsive therapy (ECT) as a safe and efficacious treatment modality which has led to remission of her PDP while DBS continues to provide adequate management of her PD symptoms. This case improves upon the early recognition of PDP and outlines a unique treatment modality not well described in the literature. This is the only case that demonstrates the efficacy of combining pimavanserin and ECT for refractory PDP in a patient with a DBS.

## Introduction

Parkinson's disease (PD) is the second most common neurodegenerative disease after Alzheimer’s disease with onset occurring at an average age of 60-65. A study from 2022 found that the prevalence of PD has increased in North America from 60,000 cases annually to 90,000, possibly due to the increasing population age, previously outdated estimates, and other environmental factors [1]. PD is a heterogeneous progressive disorder, with pathognomonic motor symptoms including rigidity, bradykinesia, resting tremors, a shuffling gait, and postural instability. In addition, Parkinson’s disease may manifest with psychiatric findings, the most common being Parkinson’s disease dementia, which affects up to 80% of PD patients. Other psychiatric manifestations include impulse control disorder and anxiety affecting up to 60%, depression affecting 35%, anhedonia affecting up to 40%, and Parkinson’s Disease Psychosis (PDP) which can develop in up to 60% of PD patients [2]. PDP in particular is associated with increased morbidity and mortality as psychosis has been identified as the primary cause of admission to nursing homes for patients with PD. PDP is characterized by vivid dreams, nightmares, and hallucinations that are typically visual and less commonly auditory. The visual hallucinations may be classified as ‘minor variants’ which include illusions such as the presence of an object or ‘formed variants’ which include the presence of people or animals [3]. While the pathophysiology behind PDP is not fully understood, it has been found that it involves a complex interplay between exogenous (drugs, intercurrent illnesses) and endogenous (neurotransmitter-dysfunction) factors [4].

The increase in prevalence of PD along with PDP occurring in a significant portion of this patient population underscores the importance of research to discover the most efficacious treatment options for PD with concurrent PDP. Initial treatment for PD includes combination pharmacotherapy such as carbidopa-levodopa which is commonly seen as the gold standard; however, in patients who fail medical therapies for PD, installation of a deep brain stimulator (DBS) is an option. Initial treatment for PDP includes dose reduction of antiparkinsonian medication, antipsychotic medication such as clozapine, pimavanserin, or quetiapine, and electroconvulsive therapy (ECT) in refractory cases. PDP occurring in patients with a DBS may also require modification of the DBS stimulus parameters. We report a 70-year-old Caucasian female with a history of diagnosed PD adequately treated with a DBS who developed PDP refractory to medication reduction, DBS stimulus adjustments and oral antipsychotics. This case report is novel for two reasons: the patient’s PDP symptoms presented only four years after her initial PD diagnosis, six years earlier than the established average [5]; the patient with a DBS was safely and efficaciously treated with combined ECT and pimavanserin - the first case reported of its kind. 

This article was previously presented as a poster at the 2023 American Psychiatric Association Annual Meeting on May 21, 2023.

## Case presentation

The patient was initially diagnosed with PD in 2011 at age 59 and was subsequently managed with carbidopa-levodopa 25-100 mg every 3.5 hours, trihexyphenidyl 2 mg twice a day, and pramipexole 0.5 mg three times a day. The patient had a history of depression and anxiety which had been stabilized for many years by her outpatient psychiatrist with escitalopram 20 mg daily, clonazepam 0.5 mg, and quetiapine 200 mg at bedtime. In June of 2015, at age 63, she presented to the emergency room with psychotic symptoms including auditory hallucinations commanding her to hurt herself along with delusions of previous coworkers telling her to take her own life. She was accompanied by her family who reported that these psychotic symptoms started in January of 2015 but were progressively getting worse. Both the patient and the family denied any associated mood symptoms such as depressed mood, decrease in energy and motivation, feeling hopeless and helpless, anhedonia, or suicidal ideation. 

Upon presentation, a routine medical workup including metabolic tests, brain imaging studies, and urine drug screen was negative. The patient was admitted to the inpatient psychiatric unit for five weeks. During her hospitalization, her psychotic symptoms did not respond to the first-line treatment modalities including dose reductions in pramipexole, trihexyphenidyl, and carbidopa-levodopa as well as an increased dosage of oral antipsychotics including quetiapine. At this point, the diagnosis of PDP was made and ECT was initiated. The patient received an acute course of ECT including nine treatment sessions during hospitalization followed by weekly out-patient maintenance sessions. Her mood was stabilized on mirtazapine 15 mg at bedtime and quetiapine 150 mg at bedtime. 

In 2016, the patient’s PD progression was unable to be managed with medication alone. Attempts at an increased dosage of her PD medication exacerbated the psychotic features she initially presented with; therefore, the patient underwent placement of left and right subthalamic nucleus deep brain stimulators (STNDBS) in 2016 and 2017 respectively at a distant referral center. Of note, ECT maintenance sessions were held during this time due to the lack of guidelines and data on ECT treatment with DBS for patients with PD and concomitant PDP. 

In 2017, the patient was readmitted to the inpatient psychiatric unit for psychotic symptoms of bizarre behavior, persecutory delusions, and auditory hallucinations. During the second hospitalization, the patient’s psychosis was again resistant to adequate trials of oral antipsychotics including quetiapine, pimavanserin, and clozapine. The patient was transferred to an out-of-state facility experienced in the management of patients with DBS requiring ECT and further adjustment of her PD and psychiatric medications. Once stabilized, the patient returned home and was able to be maintained on the same regimen on an outpatient basis. The patient is currently treated with lithium 150 mg twice a day, clonazepam 0.5 mg at bedtime, and nortriptyline 10 mg at bedtime for mood stabilization. Her PDP is treated with pimavanserin 34 mg daily and right unilateral ECT. During her acute presentation, ECT was initially conducted weekly. As her condition stabilized, we gradually shifted to every eight weeks, and the patient currently receives ECT every 12 weeks. The ECT parameters include a frequency of 70 Hz, pulse width of 0.3 milliseconds, duration of eight seconds, and current of 800 mA. PD symptoms have been well-controlled with frequent dosing of carbidopa-levodopa and Medtronic bilateral STNDBS. The patient has safely continued ECT treatments while maintaining the efficacy of her STNDBS which is deactivated before and reactivated immediately following each ECT procedure. 

## Discussion

Diagnosis

PD is well established as a degeneration of the dopaminergic neurons of the substantia nigra. This is in part attributed to the deposition of Lewy bodies composed of misfolded alpha-synuclein proteins which are commonly located in the substantia nigra, brainstem, and cortex on post-mortem microscopic analysis. This accumulation of Lewy bodies, particularly in the cerebral cortex, has been proposed to cause serotonergic neuron degeneration which ultimately leads to the upregulation of serotonergic 5HT2A receptors. Upregulation of these receptors in the prefrontal and temporal cortex is thought to significantly contribute to the symptoms of PDP which tend to develop on average ten or more years after an initial diagnosis of PD [5,6]. The uniqueness of this case lies in the early presentation of our patient's PDP, which occurred only four years after being diagnosed with PD. However, before diagnosing a patient who has underlying PD with PDP, other causes of the psychosis should be ruled out. A thorough workup might include laboratory tests for causes of delirium or exclusion of occult infection including a complete metabolic panel, complete blood count, thyroid-stimulating hormone level, liver function tests, ammonia, vitamin B12, folate levels, as well as rapid plasma reagin and urinalysis. Brain magnetic resonance imaging (MRI) should be considered for investigating other intracranial processes when psychosis is acute, persistent, and accompanied by other neurological deficits. Of particular importance in the elderly population is a thorough review of their medications to assess whether they are taking anything on the Beer’s List (medications to avoid in the geriatric population). A urine drug screen should also be conducted, regardless of patient age, to rule out a substance-induced psychosis [3]. 

This comprehensive workup conducted on our patient revealed no cause of her psychotic symptoms. Other diagnoses to consider in this patient who had an underlying history of anxiety and depression are major depressive disorder with psychotic features, schizophrenia, schizoaffective disorder, bipolar disorder, or Parkinson’s dementia with psychosis. To effectively screen for these disorders, the American Psychiatric Association details specific characteristics and timelines all of which were unrevealing for our patient [7]. Given her psychiatric history, major depressive disorder with psychotic features was a strong consideration; however, the patient denied any mood symptoms or suicidal ideation, essentially screening negative for depression upon presentation. Her cognition was intact per mental status examinations with no indication of a major neurocognitive disorder. She endorsed that her depression had been well-controlled for years on her regimen of escitalopram 20 mg daily, clonazepam 0.5 mg, and quetiapine 200 mg at bedtime. This information was corroborated by her family. Given her history of PD, PDP was then strongly considered. 

When considering PDP, one should first determine whether the psychotic features are a side effect of PD medication. Carbidopa-levodopa is a first-line medication for PD that increases the available dopamine in the CNS. While this helps mitigate the lack of dopamine caused by PD, too much dopamine can result in auditory and visual hallucinations reminiscent of the pathophysiology underlying schizophrenia; however, other PD medications such as anticholinergics and dopamine agonists are more commonly implicated. In a patient with PD presenting with psychotic features, it is recommended to proceed with lowering the dosages of PD medication. The recommended order of doing so is to, first, stop adjuvant medications for PD, including anticholinergics, selegiline, and amantadine; second, stop dopamine agonists and catechol-O-methyltransferase (COMT) inhibitors; and, lastly, reduce levodopa only if these measures fail. Of note, complete removal of all dopaminergic medications should be avoided to prevent significant worsening of Parkinsonian symptoms and, in rare instances, development of neuroleptic malignant syndrome [3]. Returning to our patient, a reduction in her PD medications was attempted; however, her PD symptoms dramatically worsened, even with her bilateral DBS. Thus, this patient was thoroughly assessed for any organic causes of her psychosis, was screened individually and through her family members for any other probable psychiatric manifestation, and was refractory to PD medication dose reduction. As such, the probable diagnosis was early onset PDP. 

Treatment

**Figure 1:**
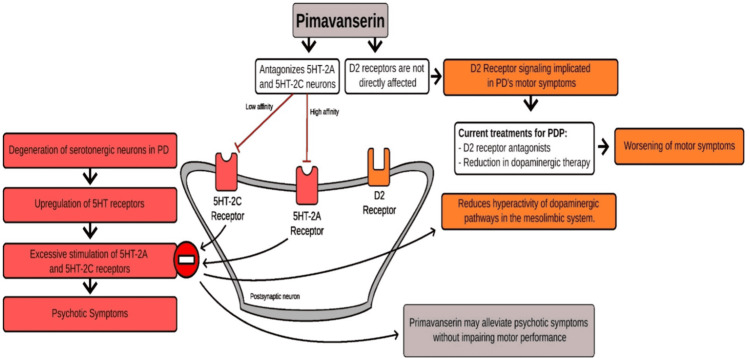
Proposed mechanism of action of pimavanserin in Parkinson’s disease psychosis. Figure reproduced from Rissardo et al. [9] in accordance with the MDPI (Multidisciplinary Digital Publishing Institute) Open Access Information and Policy.

PDP treatment is geared towards alleviating psychosis while continuing to minimize the progression of PD symptoms. For the PD component, there are the previously described pharmacologic medications and DBS, whose mechanism is to send high-frequency stimulation to specific brain regions to decrease motor sequelae of PD. The primary brain regions to target with DBS are the internal segment of the globus pallidus (GPi) and the subthalamic nucleus (STN); however, electrode placement in the STN has particularly shown a reduction in the necessary dose of additional pharmacologic agents [8]. For the PDP component, multiple antipsychotics are effective as treatment but the most utilized are quetiapine, clozapine, and pimavanserin, the latter of which has specific approval for PDP due to its unique mechanism of action. Second-generation antipsychotics such as quetiapine and clozapine antagonize 5HT2A receptors; however, they also antagonize dopaminergic D2 receptors which can exacerbate the motor symptoms of PD. Pimavanserin selectively inhibits these 5HT2A receptors without affecting D2 receptors therefore selectively treating the symptoms of PDP without worsening the motor symptoms of PD (Figure 1) [6,9].

If the psychosis is refractory to these medications, ECT is then considered. ECT is a procedure in which electrical stimulation of the brain induces a seizure. The mechanism of ECT and its therapeutic effect are not fully understood, though there are multiple theories regarding neurophysiological, neurobiological, and neuroplastic changes that are induced by the procedure. Significant research has been conducted regarding ECT-induced elevation in brain-derived neurotrophic factor (BDNF), an increase in cerebral blood flow and blood-brain barrier permeability, as well as a decrease in cortisol which all function to induce neuroplastic changes believed to ameliorate symptoms [10]. 

Aside from case reports, simultaneous treatment with both DBS and ECT has not been well documented, and no formal safety and efficacy has been studied for patients getting ECT with DBS. Furthermore, the current documented cases of joint ECT and DBS therapy have not included an individual with PDP. The concerns for safety regarding joint treatment are based on whether ECT-induced seizure activity results in shifting the DBS electrodes and causes neurological damage through direct stimulation of the DBS electrodes. Additionally, there have been cases reporting fewer rebound PD symptoms following ECT treatment the shorter the length of time that the DBS is deactivated [11]. Though these are considerations, there have yet to be documented cases of adverse events due to joint treatment with DBS and ECT supporting this as an efficacious and safe treatment option [12]. 

## Conclusions

The prevalence of PD in the United States is expected to rise in conjunction with our increased life expectancy. Psychiatric manifestations such as PDP have a significant impact on morbidity and mortality in this patient population necessitating further research into early recognition and treatment modalities. The management of a patient with PD and concomitant PDP is complex, especially when the motor symptoms are treated with a DBS. Though there have been reports demonstrating the safety and efficacy of ECT in patients with a DBS for varying neuropsychiatric diseases, we present the first use of such a modality in a patient with PDP and propose a potentially novel treatment regimen: combined maintenance ECT with pimavanserin. Healthcare workers should be made aware of these unique learning points to properly assess, guide treatment, and improve patient care. 
